# Intraoperative Lung Ultrasound in the Detection of Pulmonary Nodules: A Valuable Tool in Thoracic Surgery

**DOI:** 10.3390/diagnostics15091074

**Published:** 2025-04-24

**Authors:** Diana Yasin, Jalal Al Khateeb, Dina Sbeih, Firas Abu Akar

**Affiliations:** 1 Faculty of Medicine, Al-Quds University, East Jerusalem 20002, Palestine; diana.yasin@students.alquds.edu (D.Y.); jalal.khateeb@students.alquds.edu (J.A.K.); dina.sbeih@students.alquds.edu (D.S.); 2 Department of Thoracic Surgery, The Edith Wolfson Medical Center, Holon 58100, Israel; 3 Sackler Faculty of Medicine, Tel Aviv University, Tel Aviv 6997801, Israel

**Keywords:** lung ultrasound (LUS), transthoracic ultrasound (TUS), intraoperative lung ultrasound (ILU), lung nodules

## Abstract

In the last two decades, there has been an increased interest in the application of lung ultrasound (LUS), especially intraoperatively, owing to its safety and simple approach to detecting and assessing pulmonary nodules. This review focuses on recent advancements in intraoperative lung ultrasound in detecting lung nodules. A systematic search was conducted using databases such as PubMed and Google Scholar. Keywords included “Lung ultrasound”, “intraoperative lung ultrasound”, and “video-assisted transthoracic surgery (VATS)”. Articles published between 1963 and 2024 in peer-reviewed journals were included, focusing on the ones from the 2000s. Data on methodology, key findings, and research gaps were reviewed. Results indicated a significant advantage of intraoperative lung ultrasound (ILU) in the assessment of pulmonary nodules. ILU offers a noninvasive, real-time imaging modality that demonstrates up to 100% accuracy in detecting pulmonary nodules, with shorter time needed compared to other modalities, as well as less intraoperative periods and postoperative complications. However, some disadvantages were detected, such as operator dependency and a lack of specificity and knowledge of specific signs, as well as assisted localization via percutaneous puncture and its correct interpretation. The findings suggest that ILU has a promising future in pulmonary surgeries such as LUS-VATS but needs to be engaged more in clinical applications and modified with new techniques such as artificial intelligence (AI).

## 1. Introduction

Lung ultrasound (LUS) has emerged as a valuable diagnostic tool in pulmonary medicine; it is one of the leading modalities in lung imaging [1,2,3]. It is increasingly reducing the reliance on other imaging techniques, such as chest radiography (CXR) and computed tomography (CT) [4,5]. This rising popularity is due to various factors, including its high safety profile, as it is a noninvasive, bedside, and radiation-free method for evaluating a wide range of respiratory conditions [6]. Moreover, it provides high diagnostic accuracy, is easy to use, and is adaptable to various clinical settings, including emergency departments, intensive care units, neonatal intensive care units, and primary care, and currently, its use has even moved to intraoperative procedures to help in the localization of lung nodules [7,8,9,10]. In this article review, we aim to explore the clinical applications, diagnostic accuracy, and clinical impact of intraoperative lung ultrasound in diagnosing lung nodules. By addressing current gaps in knowledge, we hope to contribute to the broader understanding and utilization of this promising diagnostic tool in pulmonary medicine.

### 1.1. Ultrasound Evolution Through History

Ultrasound technology has evolved significantly since its development, with early research tracing back to the 19th century [11]. The concept of using sound waves for imaging was first explored in the 1820s by Christian Doppler [12,13,14,15,16]. Two of the first who started using ultrasonic waves in medicine were Karl and Friedrich Dussik. In the 1940s, they used a 1.5-MHz transmitter that worked based on the physical concept of sound attenuation that passed through the head to study the shape of the ventricles. The images produced were called “hyperphonograms” [11,17,18]. The real breakthrough in medical ultrasound occurred in the 1950s when Dr. Ian Donald adapted these sonar principles for use in diagnosing abdominal masses and swellings, focusing the most in gynecology and obstetrics, enabling the visualization of the fetus during pregnancy [18,19,20]. This led to the first clinical application of diagnostic ultrasound in the 1960s [21,22]. This was due to the launch of the first hand-held articulated arm compound contact B-mode scanner in the United States, which became one of the most popular models of ultrasound and contributed to its popularity and increasing use in therapeutic and diagnostic medicine [23]. Over time, advancements in technology, such as the development of real-time imaging and Doppler ultrasound, have expanded the use of ultrasound to areas like cardiology, musculoskeletal imaging, and emergency medicine [24,25,26,27,28].

One of the first documentations of using chest ultrasonography dates back to the 1970s in Japan [29]. In the 1990s, physicians began to use ultrasound in pulmonary medicine [30,31,32]. One of the first LUS studied signs was the “lung sliding”, which was observed by Lichtenstein et al.; they used it to exclude anterior pneumothorax, and its feasibility was 98.1% [31]. Subsequently, in 1998, Lichtenstein et al. applied LUS to distinguish between chronic obstructive pulmonary disease (COPD) and lung edema by studying the comet tail artifact sign that had a 100% sensitivity and a specificity of 92% in the diagnosis of pulmonary edema [30]. Moreover, it opened new horizons for the use of LUS in lung disease, as the examination length was only 1 min, which saved much more time than other imaging techniques. The precise year when intraoperative lung ultrasound (ILU) was first used is not clearly documented in medical literature; however, there is one study dating back to 1999 [33,34]. ILU use became more prominent with the rise of minimally invasive thoracic surgery, particularly video-assisted thoracoscopic surgery (VATS), where palpation of lung nodules is difficult. In 1998, Lesser et al. studied the implications of thoracoscopic sonography, but it could not predict the location of the nodules and was limited to peripheral nodules [35]. Though, most of research articles that discussed ILU use for lung nodule localization date from around 2010 and onward [30,35].

### 1.2. Basic Physiology of Lung Ultrasound

Ultrasound imaging, or sonography, operates on the principles of sound wave propagation and its interaction with tissues. In the process, mechanical energy above the limit of human audibility, typically in the range of 1–20 million hertz (MHz) with a wavelength of 0.6–0.01 mm, is emitted by a transducer and sent into the body. In LUS, a 3.5–5.0 MHz frequency transducer is used to visualize the pleura and subpleural structures [32,36,37].

Acoustic waves travel through different tissues at varying speeds, depending on the tissue’s density and elasticity. When the waves reach an attenuating material (e.g., muscle, fat, fluid, or tissue), some of the sound waves are reflected to the transducer as echoes, while the rest continue to/scatter; this method is called visualization by reflection [32,38]. Then, the returning echoes are converted into electrical signals, which are then processed to create an image. This is known as the time-of-flight principle, where the time taken for the sound waves to return is used to determine the depth of structures within the body [39]. The efficiency of this process relies on acoustic impedance, which is a product of the tissue’s density and sound velocity. Tissues with similar acoustic impedance result in minimal reflection, while large impedance differences lead to significant echoes, making structures like bones or air-filled cavities appear as bright areas on the ultrasound image [40,41]. The ability to capture dynamic movements, such as blood flow or heart valve motion, through Doppler ultrasound further enhances its diagnostic value in assessing vascular, cardiac, cerebrovascular, and lung conditions, as well as obstetrics [42,43,44,45].

### 1.3. Lung Ultrasound, Applications, and Transthoracic Ultrasound

LUS is implicated in the diagnosis of critically ill patients [46], as well as in chronic and acute diseases [47,48]. It is composed of five components: the thoracic wall, the diaphragm, the mediastinum, the lung parenchyma, and the pleura [5]. It visualizes the lesions in terms of shape, size, and angle (blunt or acute), in addition to the definition of the margins, echogenicity (hypoechoic or hyperechoic), and neovascularization. Doppler and contrast-enhanced ultrasound (CEUS) are used for the latter [5].

LUS can be performed supine, upright, or lateral, depending on the patient’s condition and their suspected pathology. Patients unable to lie down, such as respiratory distress patients, can undergo upright ultrasound. However, supine ultrasound is preferred for intraoperative, postoperative, and critically ill patients [47]. The right or left decubitus position is used for detecting hemothorax. For the diagnosis of pneumothorax, the patient is positioned supine, while for pleural effusion, it is best to have the patient sit upright [4,49,50,51]. Different probes can be employed, albeit the most commonly used probes are linear (5–12 MHz), curvilinear, and phased-array (2–5 MHz). The choice of the probes relies on the depth of the structures wanted to study and the habitus of the patient, as a thick chest wall will require curvilinear or phased-array probes. Higher frequency probes visualize superficial structures like pleural lines and sub-pleural space. On the other hand, lower frequency probes are preferred for deeper structures such as the diaphragm or costophrenic angle [4,47]. There are different methods of lung ultrasound scoring; nonetheless, most of them assess the presence or absence and number of two artifacts: the A lines (horizontal artifact), which are hyperechoic lines that constitute the low extravascular lung fluid and imply normal lung tissue [7,52], and B lines (vertical artifacts), a hyperechoic artifact, which is extravascular lung water and is associated with the pathological pleural plane [1,4,53]. Transthoracic ultrasound (TUS) has emerged as a critical imaging technique in the assessment of pulmonary and pleural conditions like pleural effusions, pneumothorax, thoracentesis, and drainage, and in detecting pleural and subpleural lesions and assessing peripheral lesion biopsy, making it a versatile tool in both emergency and intensive care settings. Unlike traditional radiographic methods, TUS can be performed at the bedside, enhancing diagnostic speed and patient safety [54,55,56].

### 1.4. Normal Patterns in Lung Ultrasound

Lung ultrasound (LUS) in healthy individuals typically reveals characteristic patterns that correspond to the anatomical structure of the pleura and underlying lung tissue. The pleural line appears as a smooth, hyperechoic line that moves with respiration, a phenomenon referred to as lung sliding. This movement reflects the visceral and parietal pleura gliding over each other during breathing [31]. Furthermore, in normal lung parenchyma, A-lines are seen below the pleural line. These equidistant, hyperechoic lines result from the reflection of ultrasound waves within aerated lung tissue and are indicative of the absence of pathological fluid or consolidation [1,7,57]. The normal lung field is devoid of vertical artifacts, such as B-lines [4,45,52]. Abnormal patterns in lung ultrasound (LUS) provide critical diagnostic insights into various pulmonary pathologies. One common abnormality is the presence of B-lines, which appear as laser-like lines extending from the pleural line to the bottom of the screen without fading [58,59]. B-lines are associated with increased density caused by decreased aeration of the lung periphery, such as pulmonary edema, diffuse or focal interstitial lung diseases, acute respiratory distress syndrome, or infection [53,60]. Consolidations appear as hypoechoic, tissue-like structures and are indicative of alveolar filling, exhibiting the presence of signs like comet-tail reverberation artifacts. The air/fluid bronchogram[s] is often seen in pneumonia, atelectasis, pulmonary embolism, lung cancer, and metastasis [61,62,63,64,65,66]. Pleural effusions manifest as an anechoic space between parietal and visceral pleura, usually displaying a sinusoid sign wave-like motion of the lung within pleural fluid [66,67,68]. Pneumothorax is characterized by the absence of lung sliding B-lines, lung pulse, and the presence of the “lung point”, a diagnostic sign where normal lung and pneumothorax patterns meet [66,69,70,71,72].

### 1.5. LUS and Lung Nodules

Lung ultrasound can be implemented to diagnose different cases, such as pneumonia, necrosis, abscesses, atelectasis, and pulmonary tumors. It also has a great value in diagnosing lung neoplasms [5,66]. Lung ultrasound (LUS) has emerged as a valuable tool in the evaluation of lung nodules, particularly those located peripherally [5]. Traditionally, imaging modalities like computed tomography (CT) and positron emission tomography (PET) have been employed for the assessment of pulmonary nodules [73,74]. However, LUS provides a real-time image of the lesion and whether or not the nodule moves with breathing, which facilitates the biopsy procedure and saves the patient from more invasive methods such as surgical biopsy and bronchoscopy. Furthermore, it possesses high detectability for malignant pulmonary effusions, especially small ones that are hard to detect using CXR or CT [5,29]. The definitive signs of infiltration are the expansion of the tumor into the thoracic wall and the destruction of the ribs. It is also important to assist the surrounding parenchyma for signs of vascular complications such as tumor infiltration of the vena cava or heart, metastases in the pleura and mediastinal lymph nodes, and distant metastasis in the liver [5]. Lung carcinoma is usually oval and has peripheral vascularization, with malignant vessels at the margins. Contrast-enhanced ultrasound (CEUS) can aid in biopsy and evaluate the character of the vascularization by perfusion analysis, time and pattern of enhancement, and time to wash out. Moreover, CEUS helps to differentiate necrotic from vital tumors and atelectasis from tumor tissue and should be performed before biopsy [5,75]. Doppler ultrasound can also differentiate between inflammatory and malignant lesions [76]. The resistive index (RI) is used as in other organ systems, and the higher the value, the higher the probability of a malignant lesion; the threshold is set to be 0.8 [5].

## 2. Intraoperative Lung Ultrasound (ILU) for Assistance of Lung Nodules

**Figure 1:**
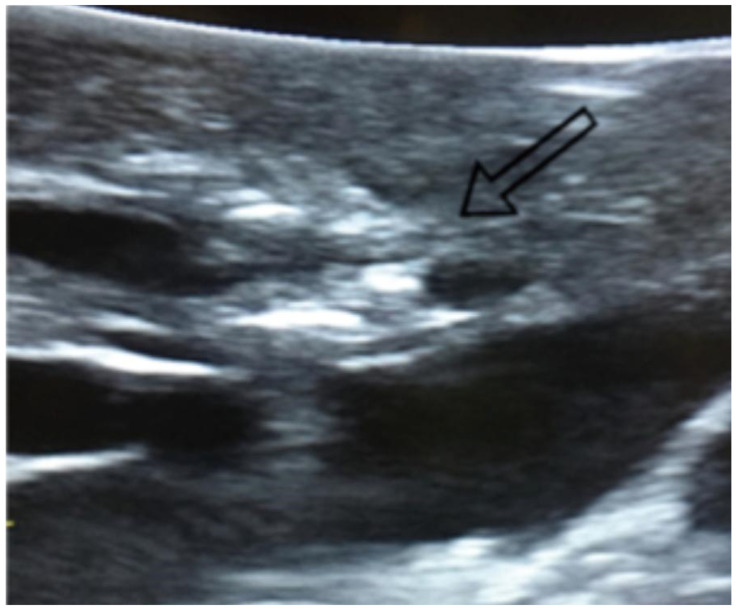
Visualization of lung nodules on intraoperative lung ultrasound. A pulmonary nodule is seen as a hyperechoic area, as indicated by the arrow.

**Figure 2:**
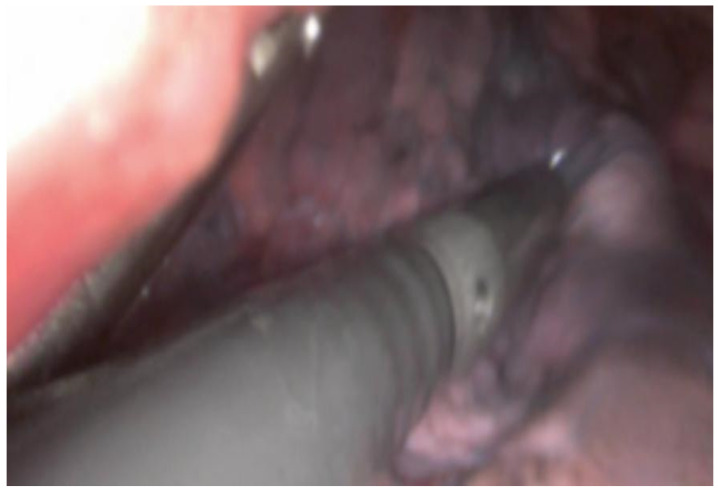
Intraoperative visualization of lung tissue during a VATS procedure. An intraoperative ultrasound probe is inserted through a VATS incision over the lung tissue to explore the lung parenchyma for pulmonary nodules.

Lung nodules are small rounded opacities in the lung tissue, measuring up to 3 cm in diameter. Larger ones are considered lung cancer until proven otherwise by histology [77,78]. Lung nodules can be classified as benign or malignant, with benign nodules being granulomas or inflammations caused by infections like tuberculosis infection or Pneumonia, or hamartomas. Meanwhile, malignant nodules may indicate early-stage lung cancer or metastatic disease [79,80]. By CT, they are classified as solid, partially solid, or non-solid (ground-glass opacities) [77]. Epidemiologically, lung nodules are common, with a prevalence of 28–30% in specific populations, influenced by risk factors such as smoking, age, ethnicity, and gender, though these results may vary widely in other populations and circumstances [81,82]. Management strategies depend on nodule size, growth rate, and patient comorbidities and preferences [83]. Surgical resection is indicated for nodules suspicious for malignancy, with video-assisted thoracoscopic surgery (VATS) being the preferred minimally invasive technique. VATS allows for precise localization and resection of small nodules, offering reduced morbidity, shorter hospital stays, and faster recovery compared to open thoracotomy. Advances in intraoperative imaging, such as lung ultrasound, further enhance the success of VATS in treating pulmonary nodules [84,85]. ILU is an innovative imaging modality that has gained significant attention for its application in the assessment and management of pulmonary nodules during surgical procedures (Figure 1). This is especially advantageous in minimally invasive surgeries, such as VATS, where palpation of the lung is limited [40,86,87,88] (Figure 2). ILU can be helpful in identifying nonpalpable pulmonary nodules on digital palpation or even during surgery, which are often small or located in challenging anatomical regions. Also, it provides high-resolution images, facilitates accurate localization of nodules, guides resection of the mass margins with minimal disruption to adjacent healthy tissue, and provides higher histological diagnostic yield [29,40]

### 2.1. Advantages of ILU

Intraoperative lung ultrasound is a relatively safe and simple approach for detecting and assessing pulmonary nodules. Several studies have investigated the accuracy and effectiveness of this modality in detecting pulmonary nodules in the last two decades. The majority of these studies shared positive outcomes and recommended more investigation [85,89,90,91,92]. The correct localization rates range between 92% and 100% among these studies. Such high detection rates were attributed to the direct contact of the US probe with lung parenchyma without any bone, air, or soft tissue border, which affects the quality of imaging and the determination of the nodule’s borders [93]. A study conducted in 2003 showed that ILU was able to localize the pulmonary nodule location in 92.5% of 40 nodules (37 nodules) in 35 patients. These nodules were already localized preoperatively by CT, PET, or both. The ILU localized eight additional nodules that had not been previously localized or visualized. The three lesions that were not localized by ultrasound have specific characteristics that prevented their detection. One was with fully calcified osteogenic sarcoma metastasis, with a cone of shadow completely indistinguishable from the artifacts from the missing sound transmission of surrounding parenchyma. Another lesion, colon carcinoma metastasis, was confused with a bronchial structure since it was hyperechoic. A third lesion, another colon carcinoma metastasis, was not detected because it was isoechoic from the surrounding parenchyma [91].

A different study was carried out in 2005, with 50 patients separated into two groups of 25 each, with one group receiving lung ultrasound and the other utilizing a radio-guided method to identify pulmonary nodules during surgery. The ultrasound identified the pulmonary nodule in 24 out of 25 patients (96%), whereas finger palpation detected it in 19 out of 25 patients (76%). In the other group, both radio-guided and finger palpation methods located the lesion in 20 out of 25 patients (80%). However, no statistical significance could be demonstrated among these different localization methods. On the other hand, this study showed that the intraoperative lung ultrasound and finger palpation methods were quicker than the radio-guided technique for nodule localization. The average duration for the ultrasound method was 8 min (ranging from 6 to 17 min), which encompassed a complete ultrasound examination of the lung; the average time for radio-guided localization was 21 min (ranging from 18 to 40 min); and lastly, the mean duration for finger palpation was 6 min (ranging from 4 to 15 min) (*p*-value < 0.05) [92].

Another study was conducted in 2019 with a relatively bigger sample of 131 patients. Some 49 patients underwent VATS, while 82 patients had open thoracotomy. Digital palpation identified 94.66% of all nodules, compared to a 100% detection rate with intraoperative lung ultrasound. Most of these nodules were smaller than 2 cm, and the size of the nodules not detected by the digital exam was smaller than that of the palpable nodules. ILU has not just proved its ability to localize nodules in this study but also to localize their margins correctly when evaluated in histology. A percentage of 95% of operating sample margins matched real margins on histology [93].

Similar results were noticed in another recent study of 33 patients where the ILU was used to locate pulmonary nodules during VATS. The nodules varied in size from 8 to 20 mm. ILU detected nodules in 97% of cases (32 out of 33), whereas palpation only detected 48.5% of nodules (*p* < 0.05). Ultrasound significantly reduced localization time compared to palpation (7.12 ± 1.87 min vs. 9.66 ± 2.62 min) (*p*-value < 0.05). This study divided the nodules into three types: solid (14 cases), pure ground-glass opacity (p-GGO, 10), and mixed ground-glass opacity (m-GGO, 9 cases). A comparison between these groups found that ILU localization was extremely effective, with a 90% success rate for p-GGO and 100% for m-GGO (*p* = 0.526). However, one nodule (10 mm in size and 10 mm below the visceral pleura) was not detected by ultrasound and palpation in an emphysema patient. CT imaging confirmed its location, and subsequent surgery revealed that it was carcinoma in situ [94].

In another study of 74 patients, forty-three patients received VATS aided with ILU (group A), and 31 patients received conventional VATS (group B). In group A, all the nodules were correctly identified 100%, while in group B the localization was correct in 96.7%, where one case localization failed, and it was necessary to enlarge the incision to about 2 cm to facilitate the insertion of the hand and the localization of the nodule by finger palpation. The time to identify the lesion was lower in group A (7.1 ± 2.2 vs. 13.8 ± 4.6; *p* < 0.05). The nodules were squamous carcinoma, adenocarcinoma, breast and colon metastases [85]. In a recent 2024 study, with a total of 64 patients, 31 (Group A) underwent wedge resection in uniportal VATS with ILU for nodule identification. The remaining 33 (Group B) patients received a wedge resection in multiportal VATS with traditional manual or instrumental palpation for the detection of the suspected lesion. The median nodule diameter was 11 (IQR 9–13) vs. 11 (IQR 9–12) mm, and the upper lobe location was 54.83% vs. 57.57%. The detection time was significantly shorter in Group A, with a median time of 9 (IQR 8–10) min versus 14 (IQR 12.5–15) min (*p* < 0.001) [90]. The variance in correct localization rates among the studies can be attributed to different factors, including the variation in sample sizes and nodules types. Another advantage of ILU use includes the lower rates of postoperative complications. This has been stated by multiple studies. The mean hospitalization rate in the Gambardella et al. study was 3.1 ± 5.3 days in the first group who received VATS aided with ILU and 3.5 ± 7.1 days in the second group who received conventional VATS (*p* = 0.287; unpaired t-test). During hospitalization, three patients (6.5%; *p* < 0.05) in the second group had air leaks that were conservatively managed, maintaining in-site chest drainage for 10 days. Otherwise, the chest tube was removed on the third postoperative day in both groups [85]. Similarly, in another study, no cases of postoperative morbidity were reported in the ILU-assisted surgeries group. While two cases of prolonged (more than 10 days) air leaks were reported in traditional manual or instrumental palpation-assisted surgeries groups, with a final postoperative morbidity of 6.1%. Both cases were managed conservatively, and patients were discharged with a Heimlich valve [90].

VATS-US can even lower the rate of VATS conversion into thoracotomy for lobectomy. VATS-US prevented the conversion of 43% of cases included in a study on 46 patients. It prevented the conversion by identifying nodules that were not identified by standard VATS and also by confirming nodule location before wedge resection, which ensured accurate resection [95]. This conversion can lead to a higher rate of complications and mortality than those who completed VATS only. So, preventing such conversion is essential and desired [96].

Additionally, ILU use lowers the mean operative time according to recent studies comparing patients who underwent ILU to those who did not. The Gambardella et al. study showed that the mean operative time was 84 ± 7.3 min in the ILU-assisted group compared to 99 ± 4 min in the other group (*p* < 0.05) [85]. Similarly, the Bastone et al. study showed that operative time was significantly shorter in the ILU-assisted group, with a median time of 33 (IQR 29–38) min vs. 43 (IQR 39–47) min in the other group (*p* < 0.001) [90].

### 2.2. Limitations of ILU

ILU has several limitations that must be carefully considered to optimize its clinical application. As with any use of ultrasound modality, a major limitation of it is operator dependency. The process of taking and analyzing ultrasound images correctly requires a high level of skill and experience, which can result in variations in diagnostic and detection accuracy. However, no specific studies aim to assess the variation in ILU use among experienced and junior thoracic surgeons since it is a new, innovative technique and is still not widely used. Therefore, misunderstanding artifacts and overlooking key findings, which can worsen patient outcomes, may occur due to the qualitative and subjective analysis of imaging artifacts [57]. An important factor that may help in reaching better outcomes is the standardization of ultrasound probes, assuring reproducible results [97]. Standardized training programs and protocols are essential to mitigate this limitation, as clinical lung ultrasound training—regardless of the type of educational method—has been proven to increase both the practical and theoretical knowledge of trainees [98].

Another limitation is that ultrasound waves are poorly transmitted through air, which poses a challenge in pulmonary imaging. Air in the lungs is a total acoustic absorber, which impairs the identification of pulmonary nodules, consolidation, and even normal anatomical structures like vessels and bronchi. Some deep nodules, however, are only detectable if the lung is fully collapsed, a condition not always achievable due to anesthetic or contraindications [89]. Although air can be manipulated, an atelectatic lung does not offer a well-organized anatomic orientation. And sometimes the pulmonary nodule is very hard to demarcate from the surrounding lung tissue since it is isoechogenic [99]. Intraoperative conditions, such as lung deflation or the presence of surgical tools, can further limit the quality of ultrasound images [100]. An innovative solution to this problem is lung liquid filling or flooding. This is performed by installing fluid like saline or PerFluoroCarbon (PFC) into the lung, which keeps it expanded. Such a procedure is used in clinical practice for other causes than lung ultrasound. While using it for lung ultrasound is still in the animal experimental phase, this technique offers a way to counteract the acoustic absorbency of the ventilated lung without the need of collapsing it. But this lung parenchyma-liquid has physical characteristics and a visual appearance that require more study and investigation to be available in clinical practice in the future [99,101,102].

While ILU is excellent for detecting general abnormalities like pleural effusion, pneumothorax, or atelectasis, its ability to differentiate between specific pathological conditions is limited. For instance, ILU cannot reliably distinguish between benign and malignant nodules or between infectious and noninfectious consolidations. This lack of specificity necessitates the use of complementary diagnostic modalities, such as computed tomography (CT), to confirm findings. Intraoperative contrast-enhanced ultrasound has emerged recently in the literature as a possible way to detect and differentiate between different malignant lung nodules using power Doppler or color-coded Doppler Sonography and contrast agent. It can help in detecting, visualizing, and characterizing these nodules by perfusion and contrast agent uptake. However, more studies still need to be performed to understand these nodules’ contrast kinetics [103,104].

### 2.3. ILU vs. Other Techniques for Pulmonary Nodule Localization

Thoracic surgeons use a technique called Assisted Localization via Percutaneous Puncture to localize pulmonary nodules. This technique depends on sophisticated tools like CT, augmented reality navigation systems, electromagnetic navigation systems, and 3D-printing technology. The localization materials consist of metallic substances, coloring agents, medical adhesives, contrast dyes, and radioactive materials. The hookwire is a very commonly used metallic substance in pulmonary nodule localization. The wire is percutaneously placed, and a portion of it is left outside the chest wall. It has a success rate range of between 97.5% and 100% [105]. This technique has notable limitations, including significant patient discomfort, a high risk of displacement or detachment, and the potential for an air embolism [106,107].

Another common technique is methylene blue dye, which was widely used in the early stages of dye-based pulmonary nodule localization. However, its rapid diffusion after injection necessitates strict surgical timing to ensure accurate localization. In patients with significant carbon particle deposition, recognizing the dye’s color can be difficult, increasing the risk of localization failure [105]. A study of 30 metastatic pulmonary nodules marked with methylene blue showed a detection rate with dyed nodules of 93% [108]. The benefits of assisted localization via percutaneous puncture encompass simple operation, brief procedural durations, broad applicability, and numerous localization choices. It also boasts a high success rate and minimal expense. Nonetheless, it carries possible complications, such as pneumothorax and bleeding, challenges in pinpointing nodules in specific areas, and the necessity to account for the surgical interval [105,108].

Another technique is the localization of pulmonary nodules via bronchoscopy; this method employs autofluorescence bronchoscopy (AFB), electromagnetic navigation bronchoscopy (ENB), and virtual-assisted lung mapping (VAL-MAP). AFB depends on indocyanine green, iodine oil, or metallic substances. It provides security, has elevated success rates of 94–100%, and has a lower cost than the electromagnetic navigation system. Nonetheless, it possesses procedural intricacy, necessitates radiation safety measures, and might entail numerous path alterations during bronchoscopy [109,110,111]. ENB utilizes dyes, metallic substances, and sensor probes to deliver real-time localization with enhanced precision and safety. The drawbacks involve expensive equipment and the requirement for skilled operators. The successful localization rate of ENB-guided localization has been reported to range from 81% to 100%. The advantage of ENB-guided localization is the lower rates of pneumothorax and hemorrhage than those achieved with CT-guided localization. However, this technique needs additional general anesthesia when ENB-guided localization is not performed in the operating room on the day of surgery [112,113,114,115,116,117,118]. VAL-MAP uses indigo carmine and metallic materials to enhance surgical margin determination. It is effective, with a successful resection rate of 98.5%, and safe, but can be costly and limited by the surgical interval [119].

As for radio frequency positioning, this technique employs microchips to tag nodules, enabling immediate identification and accurate removal, with a successful localization rate and highly successful tumor resection rates, with sufficient surgical margins of 97.5%. It is regarded as secure, yet it is restricted by significant expenses and anatomical limitations that could influence microchip positioning. Lesion removal via thoracoscopic wedge resection without radiofrequency identification marking costs EUR 4200. The radiofrequency identification delivery device costs EUR 280, while the probe costs EUR 600. As a result, wedge resection with the RFID marking system costs 1.2 times more than nonguided wedge resection [120].

Lastly, a recent technique has emerged to localize pulmonary nodules called the 3D Printing-Aided Localization technique. This creative method utilizes 3D-printed molds and substances like metals and methylene blue, or without extra materials. It has a very high localization rate of 100% according to recent two studies, but with small sample sizes. It improves accuracy in surgery, increases efficiency, and minimizes radiation exposure since it works as a substitute for a CT scan. However, accuracy is influenced by breathing and body posture, especially in individuals with a high BMI. The cost per person for creating the template ranges between USD 30 and USD 50. Compared to traditional localization, this cost will be significantly lower for patients [121,122].

## 3. Discussion

The findings from the reviewed studies highlight the significant advantages and growing potential of intraoperative lung ultrasound (ILU) in the assessment of pulmonary nodules and during surgery. ILU offers a noninvasive, real-time imaging modality that demonstrates exceptional accuracy in detecting pulmonary nodules, with localization rates ranging from 92% to 100% across the reviewed studies [85,89,90,91,92]. This high accuracy is largely due to the direct contact of the ultrasound probe with the lung parenchyma, bypassing tissue barriers.

Studies have highlighted that ILU compared to traditional methods such as finger palpation or radio-guided localization consistently demonstrated shorter detection times and higher accuracy; see for instance, Piolanti et al. (2003) and Sortini et al. (2005) [91,92]. For example, Hou et al. (2020) reported a significantly shorter localization time with ILU in video-assisted thoracoscopic surgery (VATS) (7.12 ± 1.87 min) compared to palpation (9.66 ± 2.62 min) [94]. These findings underscore the time efficiency and precision of ILU, which are critical in reducing operative time and improving surgical outcomes [91,92,94]. Additionally, ILU has proven its ability to accurately identify the margins of nodules, as shown in Chao et al. (2019), where histological evaluations confirmed a 95% match with intraoperative findings [93].

Another key strength of ILU is its association with reduced postoperative complications and shorter hospital stays. Gambardella et al. (2023) and Bastone et al. (2024) reported lower incidences of prolonged air leaks and postoperative morbidity in patients undergoing ILU-assisted surgeries compared to those relying on traditional methods [85,90]. Additionally, the reduced need for converting VATS to thoracotomy in cases of unclear nodule localization, as highlighted by Khereba et al. (2012), further emphasizes ILU’s role in minimizing surgical risks and enhancing patient recovery [2,85,95].

Despite its advantages, ILU has limitations. Operator dependency is a significant drawback, as the accuracy of ILU relies heavily on the operator’s skill and experience. The lack of standardized training protocols, artifact knowledge, and probe settings exacerbates this issue, leading to potential inconsistencies in outcomes. Furthermore, imaging deep nodules can be problematic in some conditions, such as when the lung is collapsed or deflated [89,100].

Another limitation is ILU’s inability to differentiate between benign and malignant nodules or specific pathological conditions, necessitating complementary imaging modalities like CT or PET before the surgery for staging purposes. Emerging techniques such as contrast-enhanced ultrasound hold promise for addressing this limitation, but further studies are needed to validate their clinical utility [104].

Compared to other intraoperative localization methods, ILU demonstrates clear advantages in terms of accuracy, time efficiency, and reduced postoperative complications. For instance, percutaneous puncture techniques, while effective, are associated with higher risks of complications, such as air embolisms and patient discomfort [105]. Similarly, methods like electromagnetic navigation bronchoscopy (ENB) and virtual-assisted lung mapping (VAL-MAP) require expensive equipment and specialized training, making them less accessible in resource-limited settings [100,105].

Emerging technologies such as 3D-printing-aided localization and radiofrequency positioning offer promising results but are limited by high costs and logistical challenges. In contrast, ILU provides a cost-effective, readily available, and efficient alternative that integrates easily into existing surgical procedures.

## 4. Future Directions and Conclusions

To maximize ILU’s potential, future efforts should focus on providing training programs for pulmonologists and surgeons to use the LUS efficiently on their own without supervision. This can be achieved by increasing the threshold of the scans during the training, which will minimize the misdiagnosis and misinterpretation of artifacts and signs [123,124]. Following and developing international guidelines will improve the accuracy and reliability of ILU, as by adapting the 20 statements of the use of ILU that were reviewed and updated by multidisciplinary experts from different countries in 2022 [125]. Moreover, the lack of clinical time and supervisors could be credited to the lack of knowledge and skills using LUS [126].

The matter of integrating AI in the assessment of lung cancer was also reviewed by de Margerie-Mellon et al., where they highlighted its advantage in the non-invasive characterization of tumors, histological subtypes, and somatic mutation predictions [127]. However, its use is still limited because of the lack of published studies and the enormous work needed to develop such tools [127]. Further research with larger sample sizes and diverse patient populations is also necessary to establish ILU as a gold standard in pulmonary nodule localization and assessment. In conclusion, ILU represents a valuable addition to the armamentarium of thoracic surgeons. Its high accuracy, time efficiency, and safety profile make it an indispensable tool in modern surgical practice, with the potential to revolutionize the management of pulmonary nodules. However, addressing its limitations through training and standardization will be crucial in achieving its full potential.

Overall, intraoperative lung ultrasound as a modality has a great potential in thoracic surgery generally and in pulmonary nodule detection and localization specifically. Since it is a relatively inexpensive and more affordable technique, that makes it an ideal adjunct modality for pulmonary nodules localization, especially in low-income countries. However, customized educational programs and courses can enhance doctors’ skills and accelerate the learning process. The development of this technique can not just bypass localization purposes but also help in identifying the nodule’s type.
